# 18 months follow-up of deep molecular response 4.5 (MR^4.5^) with nilotinib in patients with newly diagnosed chronic-phase chronic myeloid leukemia: a prospective, multi-center study in China

**DOI:** 10.3389/fmed.2023.1267512

**Published:** 2023-11-16

**Authors:** Bingbing Wen, Yuming Zhang, Haiqing Lin, Jin Lou, Chuangqing Tu, Yirong Jiang, Xiaolian Liu, Yan Chen, Huiqing He, Zelin Liu, Xiaoling Xie, Wangxiang Huang, Liping Pang, Xin Du

**Affiliations:** ^1^Department of Hematology, The Second People's Hospital of Shenzhen, The First Affiliated Hospital of Shenzhen University, Shenzhen, China; ^2^Department of Hematology, Affiliated General Hospital of Guangdong Medical University, Zhanjiang, China; ^3^Department of Hematology, Shenzhen People's Hospital, Shenzhen, China; ^4^Department of Hematology, Shenzhen Baoan People's Hospital, Shenzhen, China; ^5^Department of Hematology, Dongguan People's Hospital, Dongguan, China; ^6^Department of Hematology, Gaozhou People Hospital, Gaozhou, China; ^7^Department of Hematology, The Eighth Affiliated Hospital, Sun Yat-sen University, Shenzhen, China; ^8^Department of Hematology, Zhongshan City People's Hospital, Zhongshan, China; ^9^Department of Hematology, Huazhong University of Science and Technology Union Shenzhen Hospital (Nanshan Hospital), Shenzhen, China; ^10^Department of Hematology, Huizhou Municipal Central Hospital, Huizhou, China; ^11^Department of Hematology, Shenzhen Longgang Central Hospital, Shenzhen, China; ^12^Department of Hematology, Peking University Shenzhen Hospital, Shenzhen, China

**Keywords:** 18 months, deep molecular response, nilotinib, newly diagnosed, chronic-phase chronic myeloid leukemia

## Abstract

**Introduction:**

Early stable deep molecular response (DMR) to nilotinib is associated with goal of treatment-free remission (TFR) in patients with chronic-phase chronic myeloid leukemia (CML-CP). It is important to early distinguish between patients who can achieve a DMR and those who are fit for TFR.

**Methods:**

We performed a multicenter study to explore the early cumulative MR^4.5^ rate at 18 months with nilotinib in patients with newly diagnosed CML-CP (ND-CML-CP) in China. Of the 29 institutes, 106 patients with ND-CML-CP received nilotinib (300 mg BID).

**Results and discussion:**

The cumulative MR^4.5^ rate of nilotinib treatment at 18 months was 69.8% (74/106). The cumulative MMR and MR^4.0^ rates for nilotinib at 18 months were 94.3% (100/106) and 84.9% (90/106), respectively. Patients with an ultra-early molecular response (u-EMR) at 6 weeks were not significantly different in obtaining DMR or MMR by 24 months compared with those without u-EMR (*p* = 0.7584 and *p* = 0.9543, respectively). Our study demonstrated that nilotinib treatment in patients with ND-CML-CP contributed to obtain high early MR^4.5^.

## Introduction

Chronic myeloid leukemia (CML) is characterized by the presence of a BCR::ABL1 fusion gene on the Philadelphia chromosome (1). The BCR::ABL1 fusion gene produces the BCR::ABL1 tyrosine kinase, which leads to leukemia cell proliferation (2–4). Three tyrosine kinase inhibitors (TKIs) have been used for the frontline treatment of CML-CP in China including imatinib, nilotinib and dasatinib according to the NCCN guidelines (5, 6). Imatinib was first approved for therapy of newly diagnosed CML-CP (ND-CML-CP) and had efficacy superior to that of interferon-α plus cytarabine (7). Nilotinib and dasatinib, second-generation TKIs, had also been approved as therapies (8, 9). *In vitro*, nilotinib exhibited greater selectivity for ABL kinase and had a higher level of inhibitory compared to imatinib (10, 11). The recommended dose of nilotinib is 300 mg twice daily (BID) for patients with ND-CML-CP (12). Frontline nilotinib (300 mg BID) was reported to be associated with a higher rate of deep molecular response (DMR) than imatinib (MR^4.5^ by 2 years, nilotinib vs. imatinib: 26% vs. 10%; *p* < 0.0001) in the ENESTnd study (13). In addition, 54 and 61.0% of patients in the nilotinib arm (300 mg BID) achieved MR^4.5^ compared with 31 and 39.2% of patients in the imatinib arm after 5 years and 10 years of follow-up, respectively (13, 14).

Treatment-free remission (TFR) has recently become the new goal for patients with CML-CP. DMR serves as a milestone in TFR, and has been described in the European LeukemiaNet (ELN) and LALNET recommendations and NCCN guidelines (15–17). Patients with achievement of early stable DMR can acquire a chance to discontinue medication for TFR. Therefore, it is important to early distinguish between patients who can achieve of the DMR and those who are fit for TFR. However, the cumulative MR^4.5^ rate of nilotinib was not yet detected in the ENESTchina study with a 12-month follow-up, and the early cumulative incidence of the MR^4.5^ rate of nilotinib by 18 months was also not investigated in previous studies. In addition, early molecular response (EMR) may be as the first milestone in the treatment of CML-CP and a new marker for long-term outcomes such as in progression-free survival (PFS) or overall survival (OS) (17–19). Masahiro et al. showed that patients who achieved EMR at 3 or 6 months may have higher rate of DMR by 36 months and better PFS than those without EMR at 3 or 6 months with second-generation TKIs treatments (20). Therefore, we explored the relationship between ultra-early molecular response (u-EMR) including international scale (IS) decreased by more than 10% from baseline at 6 weeks and the cumulative incidence of MR^4.5^ rate in this study.

We designed a phase IV, prospective, multicenter study to detect the early cumulative incidence of MR^4.5^ rate by 18 months with nilotinib based on EUTOS long-term survival scores (ELTS) score in patients with ND-CML-CP in China. Because the cumulative MR^4.5^ rate of nilotinib was not yet detected in the ENESTchina study with a 12-month follow-up, these clinical data will be an important supplement for the efficacy of MR^4.5^ in real world ND-CML-CP patients in China. The cumulative incidences of MR^4.0^ and MMR, u-EMR, PFS and OS were also evaluated in this trial.

## Materials and methods

### Trial design and patients

This phase IV, multicenter, single-arm, prospective study was conducted in China. The key eligibility criteria included age of 18 years or above, confirmed ND-CML-CP (positive BCR::ABL1 mRNA or positive Philadelphia chromosome) within 6 months of study registration, allowed a cytoredutive therapy with hydroxyurea before starting nilotinib therapy, no accelerated phase (AP) or blast phase (BP) criteria, and an Eastern Cooperative Oncology Group performance-status (ECOG) score less than 2 were eligible for inclusion. Patients with T315I mutations in BCR::ABL1 or those who had previously received treatment with any TKI treatment were excluded. Patients with a history of severe heart or lung disease were excluded from this trial.

This study was approved by the institutional review boards of Shenzhen Second People’s Hospital and other participating institutions. This study was adhered to the ethical principles of the Declaration of Helsinki. Written informed consents was obtained from all patients prior to the study procedures. This study was registered in the Clinical Trial prs. Gov Registry (NCT03942094).

### Intervention and end points

Each patient in this trial received nilotinib 300 mg BID until disease progression. The primary endpoint was the cumulative MR^4.5^ rate at 18 months. The secondary endpoints were cumulative MR^4.5^; molecular response 4.0 (MR^4.0^); major molecular response (MMR) at 3, 6, 9, 12, and 24 months; ultra-early molecular response (u-EMR); PFS; and OS.

MMR was defined as BCR::ABL1 IS (BCR::ABL11/ABL1 ratio on the International Scale [IS]) ≤ 0.1%. MR^4.0^ was defined as BCR::ABL1 IS ≤0.01%. MR^4.5^ was defined as BCR::ABL1 IS ≤0.0032% (5). We defined the ultra-early molecular response (u-EMR) of nilotinib, including international scale (IS) decreased by more than 10% from baseline at 6 weeks, considering that previous studies defined an early molecular response (EMR) as BCR::ABL1 transcript level ≤ 10% according to the International Scale after 3 months of therapy (18, 21, 22). PFS was defined as disease progression to AP/BP or loss of response. OS was defined as the time from day 0 to the last follow-up visit or death. Adverse events owing to TKIs were determined according to the Common Terminology Criteria for Adverse Events (CTCAE) version 5.0.

### Statistical analysis

The clinical data cut-off date was September 30, 2022. Population analyses were performed using the intention-to-treat method. Data are presented as medians. The cumulative MMR, MR^4.0^, MR^4.5^, PFS, and OS were assessed using the Kaplan–Meier method, patient background, achievement of u-EMR, etc., using the log-rank test. All statistical analyses were performed using Stata software version 14.1.

## Results

### Characteristics of patients

Between July 2019 and September 2022, 106 patients newly diagnosed with CML-CP were enrolled from 29 Chinese institutions. The median age was 36 years (range, 18–76 years), and 68 patients (64.2%) were men. The median time from diagnosis to nilotinib treatment was 11 days (range, 0–84 days). The numbers of patients with low, intermediate and high ELTS scores were 72 (67.9%), 21 (19.8%), and 11 (10.4%), respectively. 5 (4.7%) patients had chromosomal abnormalities in addition to the Philadelphia chromosome, and 31 (29.2%) had a large spleen size ≥10 cm below the costal margin. The median hemoglobin (Hb), platelet count (PLT), and white-cell count (WBC) in the peripheral blood at the time of CML diagnosis were 108 g/L (range, 56–384 g/L), 451 × 10^9^/L (range, 73–3,444 × 10^9^/L) and 122.4 × 10^9^/L (range, 8.0–525.1 × 10^9^/L), respectively. Clinical characteristics and the key baseline values are summarized in Table 1.

**Table 1:** Baseline demographic and clinical characteristics of patients.

Characteristic	Nilotinib 300 mg BID (*N* = 106)
Age (y), median (range)	36 (18–76)
Male, *n* (%)	68 (64.2)
Time form diagnosis to nilotinib(days), median (range)	11 (0–84)
EUTOS long-term survival (ELTS) score, *n* (%)	
Low	72 (67.9)
Intermediate	21 (19.8)
High	11 (10.4)
Chromosomal abnormalities in addition to the Philadelphia chromosome, *n* (%)	5 (4.7)
BCR::ABL1 type, *n* (%)	
P190	0 (0.0)
P210	104 (98.0)
P190 and P210	2 (2.0)
Prior treatment with HU	71 (67.0)
Complications	
Hypertension	1 (0.9)
Diabetes mellitus	1 (0.9)
Hyperlipidaemia	5 (4.71)
Cardiopathy	0 (0)
Spleen size ≥10 cm below costal margin, *n* (%)	31 (29.2)
Median hemoglobin (range), g/L	108 (56–384)
Median platelet count (range), × 10^9/L	451 (73–3,444)
Median white-cell count (range), × 10^9/L	122.4 (8.0–525.1)

HU, hydroxyurea.

### Primary and secondary endpoints

**Figure 1:**
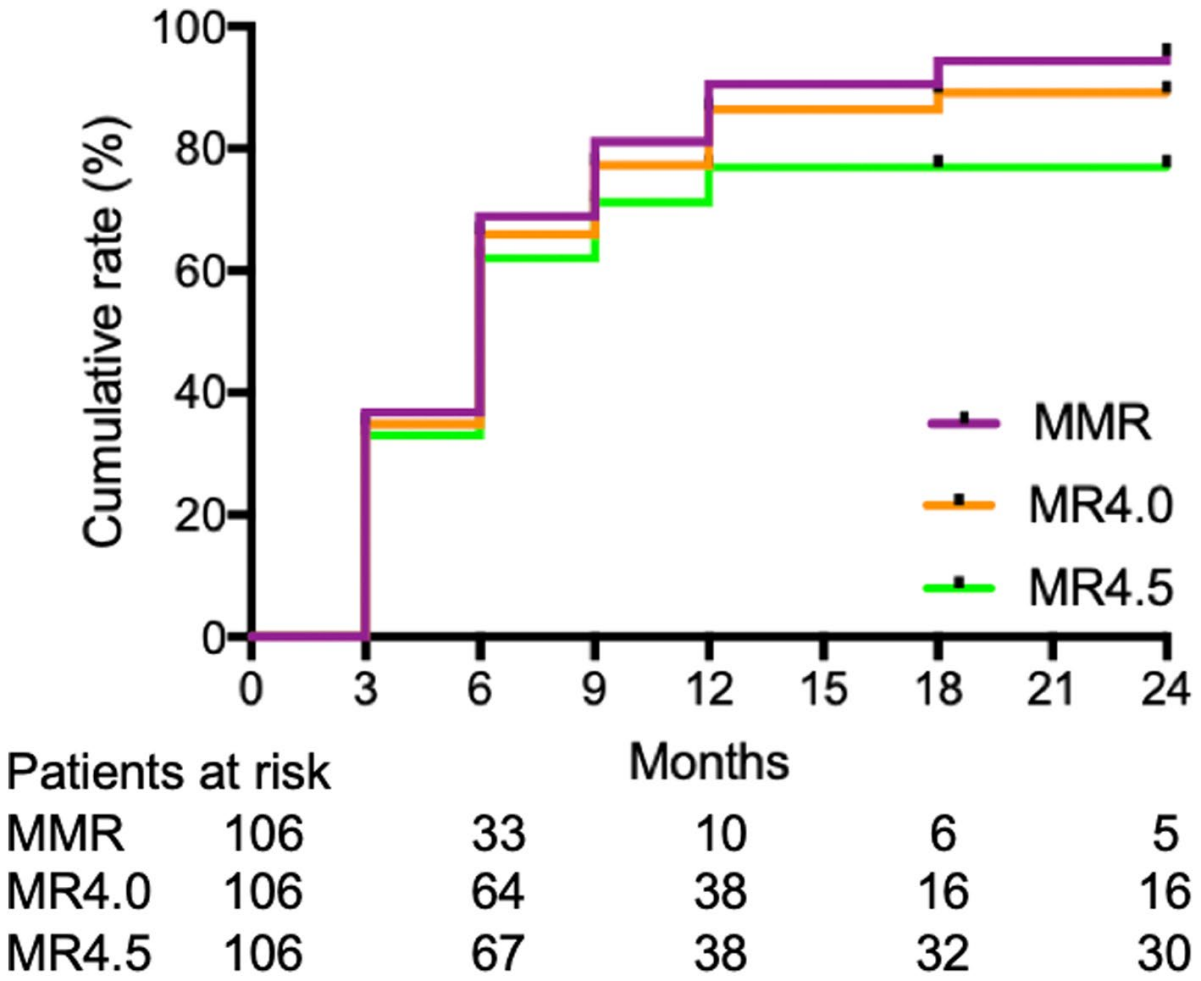
Cumulative incidence of responses of nilotinib with major molecular response (MMR), molecular response 4 (MR^4.0^) and molecular response 4.5 (MR^4.5^).

**Figure 2:**
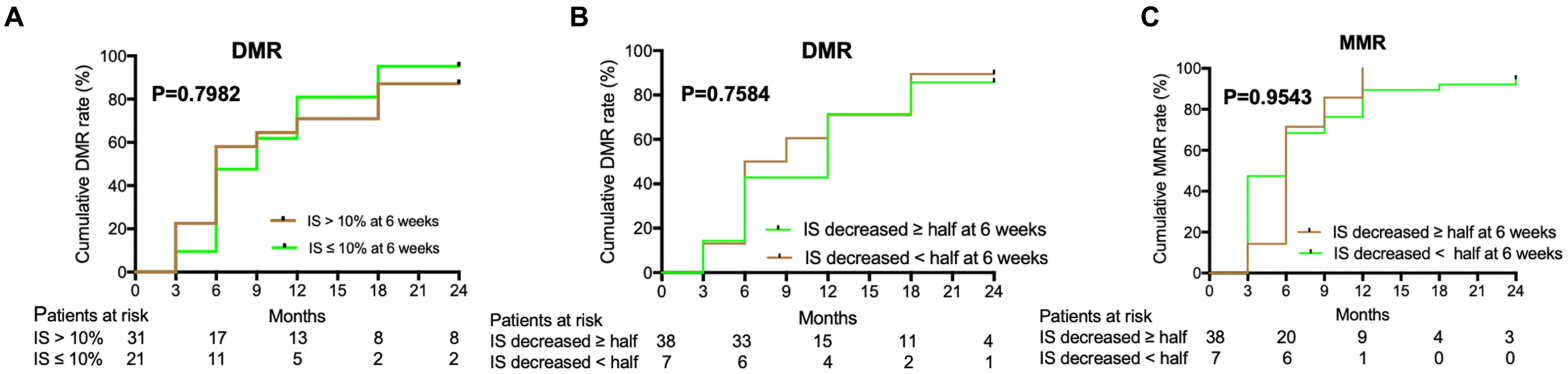
Deep molecular response (DMR) and major molecular response (MMR) were predicted by the decreased rate of IS of nilotinib at 6 weeks. **(A)** DMR of nilotinib was predicted by IS decreased >10% or IS decreased ≤ 10% of IS in baseline level at 6 weeks. **(B)** DMR of nilotinib was predicted by IS decreased ≥ half of IS or IS decreased < half of IS in baseline level at 6 weeks. **(C)** MMR of nilotinib was predicted by IS decreased ≥ half of IS or IS decreased < half of IS in baseline level at 6 weeks.

**Figure 3:**
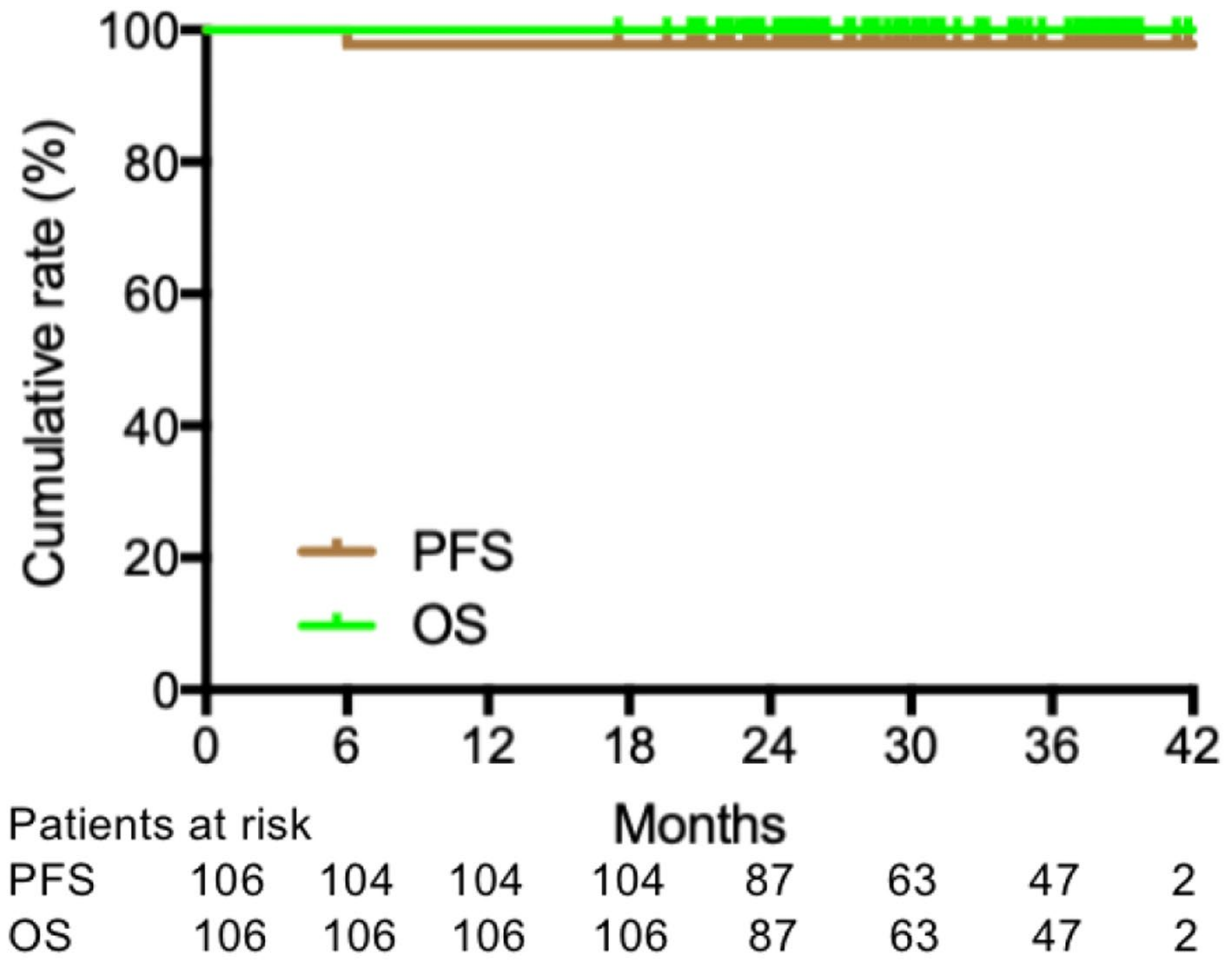
Progression-free survival (PFS) and overall survival (OS) of nilotinib.

The cumulative MR^4.5^ rate of nilotinib treatment at 18 months was 69.8% (74/106). The median time for patients to achieve MR^4.5^ was 6 months (range, 3–24) months. The cumulative MMR and MR^4.0^ rates for nilotinib at 18 months were 94.3% (100/106) and 84.9% (90/106), respectively (Figure 1). Additionally, u-EMR was achieved at 6 weeks (BCR::ABL1 IS≤10% or IS decreased ≥ half of the IS at diagnosis) by 40.4% (21/54) and 84.4% (38/45) of patients, respectively. However, patients with u-EMR at 6 weeks did not significantly differ in achieving DMR or MMR at 24 months compared to those without u-EMR (*p* = 0.7584 and *p* = 0.9543, respectively, Figure 2). The cumulative rates of PFS and OS were 98.1% (104/106) and 100% (106/106), respectively. The median followed-up period was 27 months (range, 18–42 months), and the median PFS and OS in this trial were both not reached (Figure 3).

In the univariate analysis of subgroups, patients with intermediate or high ELTS scores tended to achieve a lower cumulative response of MR^4.5^ by 18 months (HR = 0.462, *p* = 0.020; HR = 0.387, *p* = 0.042, respectively). Similarly, patients with a spleen size <10 cm below the costal margin tended to achieve a higher cumulative response of MR^4.5^ by 18 months, than those with a spleen size ≥10 cm below the costal margin (HR = 1.927, *p* = 0.021). However, there were no significant difference using multivariate analysis for nilotinib with cumulative response of MR^4.5^ before 18 months treatment (Table 2).

**Table 2:** Univariate and multivariate analysis for nilotinib with cumulative response of MR^4.5^ before 18 months treatment.

Subgroups		n/N	Univariate analysis		Multivariate analysis	
			HR (95%CI)	*p*-value	HR (95%CI)	*p*-value
Sex	Male	45/68	1			
	Female	31/38	1.461 (0.923–2.312)	0.105		
Age	Age ≤ 45	56/76	1			
	45 < Age < 60	15/23	0.872 (0.493–1.541)	0.637		
	Age ≥ 60	5/7	0.981 (0.393–2.449)	0.967		
ELTS score	Low	58/72	1		1	
	Intermediate	11/21	0.462 (0.242–0.884)	**0.020**	0.571 (0.282–1.157)	0.120
	High	5/11	0.387 (0.155–0.968)	**0.042**	0.479 (0.175–1.307)	0.150
Spleen size*	≥10 cm	16/29	1		1	
	<10 cm	57/73	1.927 (1.103–3.365)	**0.021**	1.372 (0.719–2.619)	0.494
WBC	≥10 × 10^9/L	72/102	1			
	<10 × 10^9/L	2/2	2.692 (0.648–11.180)	0.173		

Spleen size*, spleen size below costal margin; MR^4.5^, molecular response 4.5 log reduction; WBC, white-cell count; ELTS score, EUTOS long-term survival (ELTS) score; n, number; HR, hazard ratio; CI, confidence interval.In the univariate analysis of subgroups, patients with intermediate or high ELTS scores tended to achieve a lower cumulative response of MR^4.5^ by 18 months (HR = 0.462, *p* = 0.020; HR = 0.387, *p* = 0.042, respectively). Similarly, patients with a spleen size <10 cm below the costal margin tended to achieve a higher cumulative response of MR^4.5^ by 18 months, than those with a spleen size ≥10 cm below the costal margin (HR = 1.927, *p* = 0.021). There were no significant difference using multivariate analysis for nilotinib with cumulative response of MR^4.5^ before 18 months treatment.

As for TFR, in two bigger centres, 28 patients reached the condition of TFR and only 4 patients try to discontinue medication for TFR as required by themselves. RT-qPCR of BCR::ABL1 1 in 4 patients above were till negative for 6 months.

### Safety analysis

The most common hematological adverse events (AEs) were thrombocytopenia (46% [grade 3–4, 2%]), anemia (30% [grade 3–4, 4%]), leukopenia (14% [grade 3–4, 4%]), and neutropenia (13% [grade 3–4, 11%]). The most common non-hematologic AEs were rash (70%), myalgia (48%), fatigue (41%), dry eyes (37.6%), and itching (36.8%). Most patients with non-hematologic AEs were at grade 1–2 and could recover later. Besides, one patient gave up the treatment of nilotinib because of coronary heart disease. Moreover, one patient discontinued nilotinib treatment owing to high blood glucose and lipid levels (Table 3).

**Table 3:** Treatment-related adverse events (≥10%, all grades).

Adverse events	Any grade, *n* (%)	Grade 3/4, *n* (%)
Hematologic abnormality		
Thrombocypenia	46 (43.4)	2 (1.9)
Anemia	30 (28.3)	4 (3.8)
Leukopenia	14 (13.2)	4 (3.8)
Neutropenia	13 (12.3)	11 (10.4)
Biochemical abnormalities (at least 10%)		
Increased total bilirubin	61 (57.5)	5 (4.7)
Increased aspartate aminotransferase	43 (40.6)	0 (0)
Increased alanine aminotransferase	31 (29.2)	0 (0)
Increased Alkaline phosphatase	6 (5.7)	0 (0)
Hypercholesterolemia	40 (37.7)	0 (0)
Hypertriglyceridemia	5 (4.7)	1 (0.9)
Hypophosphatemia	17 (16.0)	2 (1.9)
Hyperglycemia	16 (15.1)	1 (0.9)
Hypocalcium	13 (12.3)	0 (0)
Hypopotassium	3 (2.8)	0 (0)
Hypomagnesium	2 (1.9)	0 (0)
Increased lipase	0 (0)	0 (0)
Increased amylase	0 (0)	0 (0)
QTc prolongation	5 (4.7)	0 (0)
Most frequent nonhematologic AEs (at least 10%)		
Rash	70 (65.8)	0 (0)
Myalgia	48 (45.0)	0 (0)
Fatigue	41 (38.9)	0 (0)
Dry eyes	40 (37.6)	0 (0)
Itching	39 (36.8)	0 (0)
Limb pain	31 (29.5)	0 (0)
Headache	30 (28.8)	0 (0)
Bone pain	28 (26.9)	0 (0)
Nausea	15 (14.1)	0 (0)
Loss of appetite	15 (14.1)	0 (0)
Constipation	11 (10.7)	0 (0)

## Discussion

Early stable DMR of nilotinib is associated with the goal of achieving TFR in patients with CML-CP. Therefore, it is important to early distinguish between patients who can achieve a DMR and those who are fit for TFR. Our prospective and multicenter study investigated the early cumulative MR^4.5^ rate by 18 months with nilotinib in patients with ND-CML-CP. The results of this trial showed that treatment with nilotinib contributed to a high rate of early MR^4.5^ for patients with ND-CML-CP in the real world.

Across 29 institutes in China, the cumulative MR^4.5^ rate by 18 months was 69.8% for patients with ND-CML-CP in this study, which was higher than the MR^4.5^ achieved in 24-month follow-up of the ENEST1st trial (50%) (23) and 24-month follow-up of the N-Road study (45.7%) (24) and in the Michihide’s trial with a median observation period of 3.4 years (50%) (25). In addition, the overall MR^4.5^ rates in treatments of imatinib 400 mg daily, imatinib 800 mg daily, dasatinib 100 mg daily, and nilotinib 800 mg daily groups after a long follow-up period were 57, 74, 71, and 71%, respectively (26). However, the cumulative MR^4.5^ rate has not yet been reported in the ENESTchina study with a 12-month follow-up. Frontline nilotinib 800 mg daily with a median follow-up of 78.3 months and frontline dasatinib 100 mg daily with a median follow-up of 6.5 years achieved MR^4.5^ in 75 and 79.5% of patients with ND-CML-CP, respectively (27, 28). In the future, it will be important to perform a study of frontline nilotnib 300 mg daily in patients with ND-CML-CP with long-term follow-up in China.

Similarly, for patients with ND-CML-CP, the cumulative MMR and MR^4.0^ rates of nilotinib at 18 months in this trial were higher than those in the ENEST1st and N-Road studies, both with a 24-month follow-up (94.3% vs. 80.4% vs. 82.2 and 84.9% vs. 55.2% vs. 58.3%, respectively) (23, 24). In addition, the cumulative MMR rate of nilotinib was 52.2% in the ENESTchina study with a 12-month follow-up (29). The highest MR^4.5^, MR^4.0^, and MMR rates in our study may be related to a high proportion of low ELTS scores (67.9%, 72/106), young population of CML-CP and a small body surface area (BSA) in Asian patients. Our findings suggest that nilotinib can achieve a high early DMR in patients with ND-CML-CP during TKI treatment.

DMR serves as a milestone in the process of achieving TFR, and has been described in the ELN and LALNET recommendations and NCCN guidelines (15–17). As TFR will be the new goal of CML-CP treatment in the future (30), u-EMR at 6 weeks may predict the early achievement of DMR in ND-CML-CP treatment. In our study, 84.4% of the patients with ND-CML-CP achieved u-EMR at 6 weeks, indicating that nilotinib could quickly reduce the tumor load. This may be because nilotinib has high binding affinity, high selectivity for ABL kinase, and high inhibitory activity (10, 11). Besides, Masahiro et al. reported that 87.0% (328/377) of patients with ND-CML-CP treated with nilotinib achieved EMR at 3 months (BCR::ABL1 IS <10%), which indicated a significantly superior PFS compared with those without EMR after a 5-year follow-up (*p* < 0.0001) (20). In our study, patients with u-EMR at 6 weeks were not significantly different in achieving DMR or MMR by 24 months compared to those without u-EMR. Long-term follow-up with nilotinib should be performed to identify the relationship between the u-EMR and DMR or PFS.

No patient showed exacerbation of AP/BC, and the reasons may be that nilotinib improves the poor prognosis of intermediate or high-risk patients based on Euro score, as presented in the ENESTnd trial and DASISION trials (14, 31). The cumulative rates of PFS and OS in our study were 98.1 and 100%, respectively, which are similar to the results of TARGET system study, with 94.1% PFS and 97.1% OS after a 5-year follow-up (20). Univariate analysis of the subgroups showed that patients with intermediate or high ELTS scores tended to achieve a lower cumulative response to MR^4.5^ by 18 months. Similarly, patients with a splenic size <10 cm below the costal margin tended to achieve a higher cumulative response of MR^4.5^ by 18 months, than those with a splenic size ≥10 cm below the costal margin. The may be because the intermediate or high ELTS score and larger spleen size indicate relatively high tumor loads for ND-CML-CP patients.

This study has some limitations. It was difficult to analyze the subgroups because of the small number of participants. Additionally, this study was designed as a single-arm clinical trial that lacked of comparison with other TKI treatments. Therefore, the generalizability of our results is limited. Long-term follow-up with nilotinib and comparison with other TKI treatments are required in the future studies in China.

## Conclusion

This prospective multicenter study demonstrated that treatment with nilotinib in patients with newly diagnosed CML-CP contributed to a high early molecular response 4.5 (MR^4.5^).

## Data availability statement

The original contributions presented in the study are included in the article/supplementary material, further inquiries can be directed to the corresponding authors.

## Ethics statement

The studies involving humans were approved by Shenzhen Second People’s Hospital. The studies were conducted in accordance with the local legislation and institutional requirements. The participants provided their written informed consent to participate in this study.

## Author contributions

BW: Writing – original draft. YZ: Data curation, Writing – review & editing. HL: Data curation, Writing – review & editing. JL: Data curation, Writing – review & editing. CT: Data curation, Writing – review & editing. YJ: Data curation, Writing – review & editing. XL: Writing – review & editing. YC: Data curation, Writing – review & editing. HH: Data curation, Writing – review & editing. ZL: Data curation, Writing – review & editing. XX: Data curation, Writing – review & editing. WH: Data curation, Writing – review & editing. LP: Data curation, Writing – review & editing. XD: Writing – review & editing.
